# Moving Toward Hepatitis C Elimination in Haemodialysis: HCV Antigen as a Practical Screening Alternative

**DOI:** 10.7759/cureus.108567

**Published:** 2026-05-09

**Authors:** Samantha Jingyun Koh, Wei Lun Liou, Sheryl Gan, Pei Yun Liu, Chee Chin Phang, Wan Cheng Chow, Rajneesh Kumar

**Affiliations:** 1 Gastroenterology and Hepatology, Singapore General Hospital, Singapore, SGP; 2 Nephrology, Singapore General Hospital, Singapore, SGP

**Keywords:** anti-hepatitis c virus (anti-hcv), end-stage renal failure, haemodialysis (hd), hepatitis c antigen, hepatitis c (hcv) infection

## Abstract

Hepatitis C virus (HCV) screening is routinely performed in patients undergoing haemodialysis (HD) to detect incident infections. Currently, patients undergoing HD are screened using enzyme immunoassays (EIA) that detect hepatitis C antibodies (HCV Ab) before dialysis initiation and are subsequently monitored periodically. This study evaluated the diagnostic yield of HCV core antigen (HCV Ag) testing as a screening tool in a contemporary HD population at Singapore General Hospital.

This single-centre, cross-sectional observational diagnostic evaluation included patients with end-stage renal failure on HD for more than six months who tested negative for HCV Ab by EIA. Participants underwent HCV Ag testing using the ARCHITECT assay, with a reactive threshold of ≥3.00 fmol/L. Repeat HCV Ab testing was performed after six months to assess for seroconversion.

Of 250 recruited patients, one was excluded due to known HCV infection. Among the remaining patients, none tested positive for HCV Ag, and no false-negative HCV Ab results were identified during follow-up. The study cohort included 159 males with a median age of 63 years (IQR 14) and a mean alanine aminotransferase (ALT) of 28.1 U/L.

HCV Ag testing did not identify any new HCV infections in this HD cohort, demonstrating its potential as a cost-effective and reliable alternative to periodic HCV Ab testing for routine HCV screening in this population.

## Introduction

Hepatitis C virus (HCV) infection remains a significant global health challenge, with an estimated 50 million people chronically infected worldwide according to the World Health Organization (WHO) [1]. Nearly half of these cases are reported in the Asian region [2], underscoring the urgent need for effective strategies to control and eliminate HCV in this part of the world. The advent of highly effective direct-acting antivirals (DAAs), which achieve cure rates exceeding 95%, has made the WHO's goal of eliminating hepatitis C as a public health problem by 2030 [3] an attainable target.

Patients undergoing haemodialysis (HD) are at increased risk of HCV infection compared to the general population [4,5], due to factors such as frequent blood transfusions, repeated vascular access, and potential exposure to infected dialysis equipment or units [6]. In Singapore, the prevalence of HCV infection among HD patients was reported to be approximately 2.14% [7]. Given this elevated risk, regular screening of HD patients for HCV infection is essential to identify and treat infections early, thereby reducing transmission and improving patient outcomes [8]. Current guidelines, including those from Kidney Disease: Improving Global Outcomes (KDIGO) 2022 [9] and the Singapore Ministry of Health, recommend screening HD patients every three months using immunoassays or nucleic acid testing (NAT) [10].

While enzyme immunoassays (EIA) detecting anti-HCV antibodies (HCV Ab) are widely used for initial screening, they have inherent limitations. Antibody tests cannot detect acute infections during the window period before seroconversion, and immunocompromised patients, such as those with end-stage renal failure on HD, may produce inadequate antibody responses, leading to false-negative results [11-14]. Furthermore, antibody testing cannot distinguish between past resolved and active infections [15], necessitating confirmatory NAT, which is costly and time-consuming.

HCV core antigen (HCV Ag) testing has emerged as a promising alternative screening tool for HCV [16,17]. By detecting viral proteins indicative of active infection, HCV Ag assays offer greater specificity and can identify infection earlier than antibody tests [11]. Additionally, HCV Ag testing is less expensive and has a shorter turnaround time compared to NAT, making it suitable for routine screening in populations requiring frequent testing, such as HD patients.

This study aimed to identify new cases of acute HCV infection using HCV Ag testing in a hemodialysis cohort. To evaluate the potential for missed infections, HCV EIA results were reviewed from HD records at least six months after HCV Ag testing.

## Materials and methods

Ethical approval

This study received ethical approval from the SingHealth Institutional Review Board (CIRB reference 2021/2474). Written informed consent was obtained from all participants in accordance with institutional guidelines.

Study design

This was a single-centre, observational study conducted at the haemodialysis unit of Singapore General Hospital, a tertiary academic hospital.

Study population

The total HD population at the unit during the study period from October 2021 to June 2022 comprised 670 patients. Inclusion criteria were patients undergoing HD for at least six months to allow sufficient exposure time and seroconversion window for HCV detection, reflecting dialysis-related infection risk. Patients with known HCV infection, HIV positivity, or inability to provide informed consent were excluded. Patients with HIV co-infection were excluded to reduce clinical heterogeneity and avoid ambiguity in interpretation of HCV screening results.

Screening and diagnostic algorithm

All eligible haemodialysis patients underwent initial screening for HCV infection using HCV Ag testing. Patients with reactive HCV Ag results were considered screen positive and underwent confirmatory HCV RNA testing to establish the presence of active infection. Individuals with both reactive HCV Ag and detectable HCV RNA were classified as having acute HCV infection.

Patients with non-reactive HCV Ag results did not undergo immediate HCV RNA testing. Instead, to account for the potential window period of infection and to exclude false-negative antigen results, repeat HCV Ab EIA testing was performed at least six months after the initial HCV Ag test. Patients who remained HCV Ab negative at follow-up were classified as having no evidence of HCV infection. Patients who tested HCV Ab positive at follow-up were considered to have evidence of HCV infection not detected at baseline and were excluded from baseline prevalence estimates.

**Figure 1:**
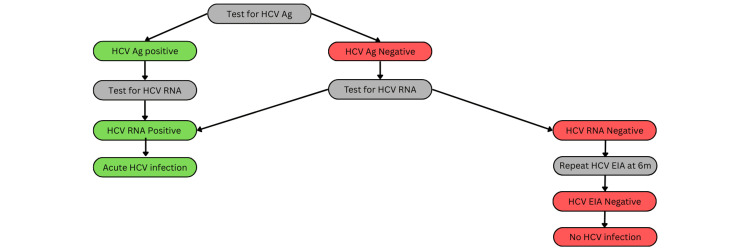
Screening and diagnostic algorithm workflow HCV: hepatitis C virus, HCV Ag: HCV core antigen, EIA: enzyme immunoassay

The hepatitis C screening and confirmatory testing pathway is illustrated in Figure 1.

Sample collection and testing

Blood samples (10 ml) were collected from participants during hospital stays, haemodialysis sessions, or routine clinical blood draws. All samples were tested for HCV Ag using the ARCHITECT HCV Ag assay (Abbott, Wiesbaden, Germany), a chemiluminescent microparticle immunoassay (CMIA). In this assay, patient samples were incubated with microparticles coated with murine anti-HCV antibodies to capture HCV Ag if present. Subsequently, acridinium-labeled murine anti-HCV antibodies were added to bind the captured antigen. The chemiluminescent signal generated upon reaction with Pre-Trigger and Trigger solutions was measured and directly correlated with the HCV Ag concentration using a calibration curve. 

For samples reactive on HCV Ag testing, confirmatory HCV RNA quantification was performed using the Cobas AmpliPrep/Cobas TaqMan HCV Quantitative test v2.0 (Roche CAP/CTM; Basel, Switzerland) per manufacturer instructions. RNA extraction was conducted from 720 μL aliquots using the automated Cobas Ampliprep instrument, followed by amplification and detection.

Follow-up

Patients underwent repeat HCV Ab EIA testing at least six months after initial HCV Ag testing to identify any missed infection.

Interpretation of results

An HCV Ag concentration <3.00 fmol/L was considered non-reactive, and an HCV Ag concentration ≥3.00 fmol/L was considered reactive.

For samples with reactive values between 3.00 and 10.00 fmol/L, duplicate retesting was performed. If both retest values were non-reactive, the specimen was classified as non-reactive. If one or both duplicates were reactive (≥3.00 fmol/L), the specimen was considered repeatedly reactive, and the initial value was reported as the final result.

Statistical analysis

The study sample size was determined by the number of eligible haemodialysis patients available during the study period. Given an expected HCV prevalence of approximately 2% from prior literature, a sample of 252 patients provides an estimated precision of ±1.8 percentage points at the 95% confidence level, which was considered acceptable for prevalence estimation in a single-centre HD population.

Descriptive statistics were used to summarize patient demographics and test results. Continuous variables were presented as means or medians with interquartile ranges as appropriate. Categorical variables were expressed as counts and proportions. No formal hypothesis testing was conducted due to the descriptive nature of the study.

## Results

Study population and demographics

**Figure 2:**
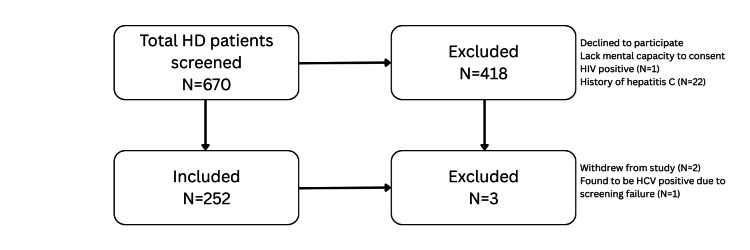
Inclusion and exclusive criteria HCV: hepatitis C virus, HD: haemodialysis

Between October 2021 and June 2022, a total of 670 patients receiving haemodialysis at Singapore General Hospital were screened for inclusion in the study. After applying exclusion criteria, including refusal or inability to consent (395 patients), HIV positivity (one patient), and known HCV infection (22 patients), 252 patients were enrolled for HCV Ag testing. Two patients subsequently withdrew, and one was excluded due to screening failure, resulting in 249 evaluable participants (Refer to Figure 2). The cohort consisted of 159 males (63.9%) with a median age of 63 years (interquartile range [IQR] 14). Baseline alanine aminotransferase (ALT) data were available for 201 participants. ALT levels were generally low, with a median ALT of 16 U/L (IQR 11-25 U/L) and an overall range of 5-108 U/L. Using an upper limit of normal of 40 U/L, 180/201 participants (89.6%) had ALT values within the normal reference range.

HCV prevalence in the haemodialysis unit

In the current study, 23 patients with established HCV infection were identified and excluded before enrolment. This corresponds to an estimated HCV prevalence of approximately 3.4% within the haemodialysis population during the study period, which is broadly in keeping with previously published local data [7].

HCV Ag screening results

Among the 249 haemodialysis patients without known HCV infection who underwent HCV Ag testing, none had a reactive result. All samples yielded HCV Ag concentrations below the reactive cutoff of 3.00 fmol/L, and no samples required confirmatory HCV RNA testing. These results indicate that no new active HCV infection was detected in the screened cohort during the study period, consistent with a low-incidence setting. However, the absence of reactive cases limits any direct assessment of the diagnostic performance of HCV Ag testing in this study.

Follow-up serological testing

To assess the potential for missed infections due to the window period or false-negative results, repeat HCV Ab EIA testing was performed at least six months after initial HCV Ag testing. No cases of seroconversion or false-negative HCV Ab results were identified during follow-up, supporting the reliability of the initial screening strategy.

Summary

Overall, HCV Ag testing did not identify any new active HCV infections in this screened haemodialysis cohort during the study period. Follow-up HCV antibody testing did not identify seroconversion among participants with available follow-up data. These findings are consistent with a low-incidence, well-controlled setting and support further evaluation of HCV Ag testing as a practical screening tool for new active HCV infection in haemodialysis populations.

## Discussion

Our study evaluated the use of HCV Ag testing as a screening tool for detecting new active HCV infection in an HD cohort during the study period, within a low-incidence, well-controlled setting. In this screened cohort of haemodialysis patients without known HCV infection, no new incident active HCV infection was identified by HCV Ag testing. However, as patients with known HCV infection were excluded, this study does not determine the overall prevalence of active HCV infection in the entire haemodialysis unit. Follow-up repeat HCV Ab EIA testing at six months did not identify seroconversion among the screened participants with available follow-up data. However, this finding should be interpreted cautiously given the small sample size, exclusion of patients with known HCV infection, and the potential for selection bias related to non-participation. 

ALT values in this cohort were generally within the low-to-normal range. However, these findings should be interpreted cautiously, as ALT alone cannot be used to exclude active HCV infection, particularly in haemodialysis patients. The demographic and clinical characteristics of the screened participants appeared broadly similar to those of the institutional haemodialysis population; however, the generalizability of these findings remains limited by the substantial non-participation rate and potential selection bias.

HCV Ag testing offers several advantages over traditional antibody-based screening in haemodialysis patients. Unlike antibody assays, which cannot differentiate between resolved and active infections and may fail to detect acute infections during the serological window period, HCV Ag testing directly identifies viral proteins indicative of active replication. This distinction is particularly critical in immunocompromised patients, such as those undergoing haemodialysis, who may exhibit delayed or diminished antibody responses [11,12]. Multiple studies have demonstrated high sensitivity (90-99%) and specificity (~100%) of HCV Ag assays, with strong correlation to HCV RNA levels, validating their role as a practical surrogate marker for active infection [16-19].

These findings are consistent with prior research demonstrating a strong correlation between HCV antigen levels and HCV RNA viral load, reinforcing the diagnostic value of HCV Ag testing. Specifically, the ability of HCV Ag to detect HCV infection correlates strongly with HCV RNA levels. This relationship is supported by a systematic review by Freiman et al. [20], which demonstrated a robust correlation between HCV Ag and HCV RNA in patients with RNA loads exceeding 3000 IU/ml. Similarly, Florea et al. [21] reported a positive correlation between HCV Ag positivity and increasing RNA loads: 17.6% positivity when RNA was below 1000 IU/ml, 88% positivity between 1000 and 10,000 IU/ml, and 100% positivity above 10,000 IU/ml. However, false-negative results where HCV Ag failed to detect active infection despite RNA levels above 3000 IU/ml have been observed, potentially due to genotypic variability and co-infections with hepatitis B or HIV [22,23]. These findings highlight both the utility and the limitations of HCV Ag testing in clinical practice.

Hence, a more reliable method to test for active HCV infection in the haemodialysis population is necessary. In a previous study by Kumar et al. [24], using HCV RNA as the marker for current HCV infection, HCV Ag testing was positive in 19 acute HCV infections who had HCV Ab that were negative. The study reported a sensitivity of 98.4% (HCV Ag assay detected 61 out of 62 positive samples) and a specificity of 100% (27 true negative samples) in detecting active acute and chronic HCV infections. Wong et al. [19] also demonstrated a similar sensitivity of 90.7% and specificity of 100% in 112 patients with anti-HCV seropositive samples, while Wasitthankasem et al. [25] reported 99% sensitivity and 100% specificity. Furthermore, in a larger study of 5,394 samples testing negative for anti-HCV antibodies, the specificity of the HCV Ag assay in HCV RNA-negative samples was established to be up to 99.98% [26].

From a cost-effectiveness perspective, HCV Ag testing presents advantages over NAT and antibody assays [27,28]. It requires less expensive reagents, simpler laboratory infrastructure, and personnel time, with shorter turnaround times, making it suitable for routine biannual screening protocols in HD patients who require frequent monitoring due to ongoing exposure risks. In low-prevalence settings like ours, where active HCV infection burden is minimal, HCV Ag testing may optimize resource allocation without compromising diagnostic accuracy.

HCV Ag testing may offer practical advantages in screening for active infection, but our study was not designed to compare screening strategies or assess false-positive reduction; therefore, further comparative studies are needed before changes to existing HD screening protocols can be recommended. Compared with antibody-based assays, HCV Ag testing may provide a more direct means of identifying active infection and may offer operational or cost advantages in certain settings. However, whether this translates into earlier intervention, reduced transmission risk, or more efficient resource utilization was not directly evaluated in our study. Furthermore, the single-centre design and modest sample size limited detection of very rare infections and may constrain generalizability. As confirmatory HCV RNA testing was performed only for reactive HCV Ag samples, low-level viraemia below the antigen detection threshold could have been missed. Future multicentre studies with larger cohorts and parallel RNA testing are needed to validate these findings and refine screening algorithms.

## Conclusions

In conclusion, HCV Ag testing is a reliable alternative to periodic antibody testing for routine HCV screening in haemodialysis patients within low-prevalence, well-controlled settings. Adoption of this approach could enhance HCV surveillance, facilitate earlier detection of active infections, and support ongoing elimination efforts in this vulnerable population.
